# Reliability and validity of the Chinese version of the Psoriasis Disability Index (PDI) in Chinese patients with psoriasis

**DOI:** 10.1186/1477-7525-10-37

**Published:** 2012-04-13

**Authors:** Zehui He, Chuanjian Lu, Aihua Ou, Jiqian Fang, Dongmei Wang, Jingwen Deng, Zhongzhao Zhang, Jingjie Yu

**Affiliations:** 1 Department of Medical Statistics and Epidemiology, School of Public Health, Sun Yat-sen University, 74 ZhongShan Er Road, Guangzhou, 510080, China; 2 Department of Dermatology, the Second Clinical College, Guangzhou University of Chinese Medicine, Guangzhou, 510120, China; 3 Department of Clinical Epidemiology, the Second Clinical College, Guangzhou University of Chinese Medicine, Guangzhou, 510120, China

**Keywords:** Psoriasis, Quality of life, Chinese, Psoriasis Disability Index, Exploratory factor analysis

## Abstract

**Background:**

The Psoriasis Disability Index (PDI) is a widely used instrument to measure the impact of psoriasis on patients. There has not been psychometric evaluation of the Chinese version of PDI. The aim of this study was to evaluate its reliability and validity among Chinese patients with psoriasis.

**Methods:**

A multi-center, cross-sectional study was conducted at 9 hospitals including patients aged 18 years and over. Reliability was determined by internal consistency using Cronbach’s alpha. Validity was assessed through convergent validity and known groups validity. Dimensionality of the PDI was examined by exploratory factor analysis in working patients and nonworking patients respectively.

**Results:**

In all, 831 patients were studied. Internal consistency of the PDI was satisfactory. Cronbach’s alpha coefficient was 0.91 for the total score and over 0.70 for each subscale of the PDI. Evidence of convergent validity of the PDI was proved by excellent and moderate to good correlations with the Dermatology Life Quality Index (DLQI) and four subscales of the Short Form-36 (SF-36) (role-physical, bodily pain, social functioning, and role-emotional): *r* = 0.51-0.78. Known groups validity was confirmed that the PDI score discriminated well among patients with different severity of psoriasis. The dimensionality of the PDI was determined by the presence of two-factor structure for working patients and three-factor structure for nonworking patients which accounted for 57.3% and 62.3% of the variance respectively.

**Conclusion:**

The Chinese version of the PDI is a reliable and valid instrument to assess the impact of psoriasis on patients’ lives and could be used in future quality of life assessment of Chinese patients with psoriasis.

## Background

Psoriasis is a chronic, systemic, and incurable skin condition characterized by the skin signs of thick scaling red plaques with itching and arthritis. It has been proved that psoriasis is associated with risk of cardiovascular disease, diabetes, overweight/obesity and depression [1-5]. Psoriasis can has profound effects on patients’ daily living and functioning [6-8]. While measurements of clinical severity separately using tools such as the Psoriasis Area and Severity Index (PASI) [9] are not sufficient and may not reflect patients’ own perceptions of the impairment due to psoriasis, it is important to assess the impact of psoriasis on patients’ physical condition, self-perception, and social life [10,11]. Health-Related Quality of Life (HRQOL), covering almost all these parameters, is therefore increasingly part of the clinical research and practice. HRQOL assessment can provide valuable information that helps make clinical decision and select suitable health care programmes [12,13].

There has been a wide variety of generic, dermatology and psoriasis-specific instruments used for the assessment of HRQOL of psoriasis patients [14-18]. However, the reliability and validity of these instruments are not fully known [19-21]. The reliability and validity are the major evaluations of instruments’ performance of reflecting concepts or ideas such quality of life (QOL) in a study population [22]. Evaluating the characteristics of instruments used to measure patients’ perceptions is important in clinical health care and decision making.

The Psoriasis Disability Index (PDI) [15], as a psoriasis-specific instrument, was one of the attempts to measure and quantify the impact of psoriasis on patients’ daily lives . It has been used internationally for almost 20 years and has been translated into at least 13 languages [23]. The reliability and validity of the PDI have been evaluated using different languages and in different psoriasis populations. However, the Chinese version of PDI has not been formally validated in Chinese patients with psoriasis. In China, HRQOL research has made a remarkable progress in patient populations [24], but studies on patients with psoriasis at a population level are limited. One of the main reasons is a lack of suitable instruments in Chinese.

In this study, permission was sought and thereby given from the developers of the PDI. Then we used psychometric methods to evaluate the reliability and validity of the Chinese version of PDI in patients with psoriasis.

## Materials and methods

### Study design and subjects

A multi-center, cross-sectional study of patients with psoriasis in 9 large hospitals was performed from November 2010 to April 2011. For a representative sample of Chinese patients with psoriasis, outpatients and inpatients with psoriasis were recruited consecutively in dermatological clinics of the 9 hospitals located in different geographical regions of China. In the north and south of China, respectively two hospitals participated in the study. In the east and middle of China, respectively one hospital participated in the study. In the west of China, three hospitals participated in the study. Inclusion criteria for this study were: (1) a clinical confirmed psoriasis diagnosis; (2) age 18 years or older; and (3) willingness to provide consent to participate. Patients were excluded if they had a severe mental illness.

After the purpose and the contents of the study had been fully explained to the subjects, written informed consent was obtained. Then all participants were asked to fill out the questionnaires according to their own feelings and opinions. Those who have difficulties in completing questionnaires by themselves finished the questionnaires with the help of trained investigators, who have been trained that every answer should be solely based on participant’s own response. Completed questionnaires were collected as soon as they were finished.

This study was approved by the Ethics Committee of the Guangdong Provincial Hospital of Traditional Chinese Medicine.

### Measurement tools

The measurement tools consisted of PDI, Dermatology Life Quality Index (DLQI), Health Survey Short Form (SF-36) and PASI. The DLQI and SF-36 were used to evaluate the convergent validity of the PDI. The PASI, frequently as an assessment of the severity of psoriasis, was used to evaluate the known groups validity of the PDI.

#### *Psoriasis disability index (PDI)*

The PDI, developed by Finlay and Coles, concerns the functional lifestyle disabilities caused by psoriasis [15]. It contains 15 items with 5 subscales: daily activities, work, personal relations, leisure, and treatment. All items are rated on a 4-point scale, with responses of “not at all”, “a little”, “a lot”, and “very much” scored 0, 1, 2, and 3, respectively. Item scores are summed to yield a total score (range: 0–45) with higher score indicating greater limitations experienced because of psoriasis. Particularly, the PDI has a possible 5 work items of which only 3 items need to be responded. Respondents who are working (either full- or part-time) respond to item 6a, 7a, and 8, whereas respondents who are not working respond to items 6b, 7b, and 8. When one item of the PDI was not responded, it was scored “0”. When two or more items of the PDI were not responded, the questionnaire was excluded from the analysis.

#### *Dermatology life quality index (DLQI)*

The DLQI, developed by Finlay and Khan, assesses the QOL impact of skin disease [14]. Comprising 10 items, the DLQI has been used internationally for more than 15 years and translated into more than 80 international languages [25,26]. Each item is scored on a 4-point scale (range 0–3). The final score on the questionnaire is the sum of the score of each item (range 0–30). Higher scores indicate worse QOL. Its Chinese version has been evaluated and shown good psychometric properties [27].

#### *Short form-36 (SF-36)*

The SF-36 is a measure of health status and is commonly used in clinical and health services research [28]. Its Chinese version has been tested for psychometric properties [29]. It consists of 8 subscales: physical function (PF), role-physical (RP), body pain (BP), general health (GH), vitality (VT), social functioning (SF), role-emotional (RE), and mental health (MH). The scores of the 8 subscales are calculated according to the scoring algorithm of the SF-36 user’s manual. The higher score, the better health. .

#### *Psoriasis area and severity index (PASI)*

The PASI assessed both intensity and extent of the psoriatic plaques separately for four anatomical regions (head, trunk, upper and lower extremities) by the physician. The PASI score ranges from 0 (no psoriasis) to 72 (very severe psoriasis), and it was normally regrouped into three categories implying three severity levels of psoriasis: PASI 0–7 (mild severity), PASI 7–12 (moderate severity), and PASI 12–72 (severe severity) [30].

Furthermore, demographic and psoriasis-specific variables such as age, gender, marital status, education, co-existing chronic disease, family history of psoriasis, duration, and clinical type of psoriasis, were reported.

### Statistical analysis

For each subscale of the PDI, the floor and ceiling effects were assessed. If more than 20% of the patients reported lowest or highest possible score, the floor or ceiling effects exist [31]. Cronbach’s alpha and item-total correlations were determined to assess the internal consistency. Construct validity, which hypothesizes a scale measure or correlate with the theorized psychological scientific construct that it purports to measure, was assessed by convergent validity and known groups validity. Convergent validity assessed the degree to which the PDI was similar to (converged on) other measures that it should theoretically be associated to. Spearman’s rank correlation coefficient was used to assess correlations between the PDI and the DLQI, the SF-36, which are the two potential measures that assess similar underlying phenomenon as the PDI. Known groups validity tested the ability of the PDI to discriminate between groups that differed in the severity of psoriasis (assessed by PASI). The Kruskal-Wallis test was used to test for statistical differences between the groups. Dimensionality of the PDI was analyzed for patients who were working and not working separately because the work items of the PDI were different for working and nonworking patients. Exploratory factor analysis was used by means of principal component analyses. Oblique rotation (promax) was performed according to the supposed correlations between the factors.

All statistical analysis was performed using SPSS 17.0 (SPSS Inc., Chicago, IL, USA).

## Results

### Subjects

In total, 884 patients participated in this study. Out of these, 9 patients were less than 18 years old. Further, 44 patients were excluded because they had two or more missing answers on the PDI. Hence, the analysis was carried out on a total of 831 patients (94%).

At study inclusion the mean age of patients was 38.6 years (SD = 13.8) and a higher proportion of patients were males than females (62% vs.38%). cardiovascular, musculoskeletal, gastrointestinal, anaphylactic, and gynecological diseases. Twenty-three percent of patients had a family history of psoriasis. The mean duration of psoriasis was 9.3 years (SD = 9.0). The most common clinical type of psoriasis was psoriasis vulgaris (92%), followed by erythrodermic psoriasis (4%), psoriatic arthritis (3%), and pustular psoriasis (1%). The mean severity of psoriasis assessed by PASI was 11.6 (SD = 10.6). Further demographic and clinical characteristics of the patients from different geographical regions are shown in Table 1.

**Table 1:** Demographic and clinical characteristics of the subjects

	**Regions**					**Total sample(*N* = 831)**
**Characteristics**	**North (*N* = 172)**	**South (*N* = 180)**	**East (*N* = 96)**	**West (*N* = 284)**	**Middle (*N* = 99)**	
Gender (%)						
Male	101(58.7)	119(66.1)	55(57.3)	164(57.7)	74(74.7)	513(61.7)
Female	71(41.3)	61(33.9)	41(42.7)	120(42.3)	25(25.3)	318(38.3)
Age (%)						
18-30 years	65(37.8)	61(33.9)	25(26.0)	113(39.8)	24(24.2)	288(34.7)
31-45 years	49(28.5)	64(35.6)	31(32.3)	117(41.2)	46(46.5)	307(36.9)
46-60 years	44(25.6)	39(21.7)	25(26.0)	35(12.3)	23(23.2)	166(20.0)
60 years	14(8.1)	16(8.9)	15(15.6)	19(6.7)	6(6.1)	70(8.4)
Marital status (%)						
Married or living with partner	111(64.5)	126(70.0)	70(72.9)	196(69.0)	76(76.8)	579(69.7)
Single	55(32.0)	50(27.8)	22(22.9)	84(29.6)	22(22.2)	233(28.0)
Separated/divorced	3(1.7)	2(1.1)	4(4.2)	3(1.1)	1(1.0)	13(1.6)
Widowed	3(1.7)	2(1.1)	0(0.0)	1(0.4)	0(0.0)	6(0.7)
Level of education (%)						
None	0(0.0)	3(1.7)	1(1.0)	0(0.0)	1(1.0)	5(0.6)
Primary school	8(4.6)	13(7.2)	1(1.0)	30(10.6)	3(3.0)	55(6.6)
Secondary school	75(43.6)	85(47.2)	40(41.7)	109(38.4)	36(36.4)	345(41.5)
College	83(48.3)	72(40.0)	52(54.2)	139(48.9)	56(56.6)	402(48.4)
Higher than college	6(3.5)	7(3.9)	2(2.1)	6(2.1)	3(3.0)	24(2.9)
Other chronic disease (%)	26.2	31.7	26.0	29.2	19.2	27.6
Family history of psoriasis (%)	25.0	22.2	26.0	19.0	26.3	22.6
Duration in years mean (SD)	10.3(11.0)	8.0(7.0)	11.0(10.0)	8.4(8.8)	11.3(7.1)	9.3(9.0)
PASI score mean (SD)	11.0(9.5)	11.0(10.2)	9.8(8.6)	12.0(11.8)	14.0(10.6)	11.6(10.6)

### Response distribution

All subscales of the PDI had small ceiling effects (<6%) but mild floor effects (>20%) for work, personal, and treatment (Table 2).

**Table 2:** Descriptive information and Cronbach’s alpha of the PDI total scale and its subscales

	**No. of items (score range)**	**Mean**	**SD**	**Percentile**			**% floor**	**% ceiling**	**Corrected item-total correlation**	**Cronbach's *α***
				**25**	**50**	**75**				
Chinese version										
Daily activities	5(0–15)	5.3	3.5	3	5	8	5.2	1.7	0.58-0.70	0.82
Work	3(0–9)	2.5	2.5	0	2	4	27.9	4.5	0.62-0.72	0.86
Personal	2(0–6)	1.3	1.6	0	1	2	30.4	4	0.64-0.65	0.79
Leisure	4(0–12)	2.9	2.6	1	2	4	17.9	0.8	0.32-0.65	0.74
Treatment	1(0–3)	0.7	0.9	0	1	1	34.7	5.7	-^a^	-^a^
Total	15(0–45)	12.8	9.4	6	11	18	3.1	0.5	0.37-0.74	0.91
English version ^b^										
Total score	15(0–45)	7.3	7.2	2	-	11	14.7	0	-	-

^a^ Scale consists of one item.

^b^ From a validation study done in the United States [32].

### Reliability

The means, standard deviations, percentiles, corrected item-total correlations, and Cronbach’s alpha coefficients of the PDI and its subscales are presented in Table 2. The subscales of PDI showed Cronbach’s alpha coefficients above 0.7, and the item-total correlation ranged from 0.32 to 0.74, which suggested an adequate internal consistency of the PDI [32,33].

### Convergent validity

Convergent validity was assessed as satisfactory. The correlations between the total PDI score and the DLQI, SF-36 are shown in Table 3. Correlations from 0.25 to 0.50 suggest a fair degree of relation; those from 0.50 to 0.75 are moderate to good; and values greater than 0.75 are considered good to excellent [34]. Results in Table 3 showed excellent correlation between the PDI and the DLQI, moderate to good correlations between the PDI and four subscales of the SF-36 (RP, BP, SF, and RE), and fair correlations between the PDI and the other subscales of the SF-36. There were negative correlation coefficients between the PDI and SF-36 score, because higher PDI score indicated greater functional lifestyle disabilities, whereas higher SF-36 score indicated better health or performance.

**Table 3:** Spearman’s rank correlation coefficients between the total PDI score and the DLQI, SF-36 scores

	***N***	***r*_*s*_(*P*)**
DLQI	815	0.78 ^a^ (<0.001)
SF-36		
Physical functioning	800	−0.42 ^c^ (<0.001)
Role-physical	818	−0.58 ^b^ (<0.001)
Bodily pain	822	−0.51 ^b^ (<0.001)
General health	803	−0.34 ^c^ (<0.001)
Vitality	809	−0.40 ^c^ (<0.001)
Social functioning	814	−0.65 ^b^ (<0.001)
Role-emotional	826	−0.58 ^b^ (<0.001)
Mental health	796	−0.45 ^c^ (<0.001)

^a^ Excellent correlation. ^b^ Moderate to good correlation. ^c^ Fair correlation.

*N*: Sample size when correlation was calculated. *r*_*s*_: Spearman’s rank correlation coefficients.

### Known groups validity

**Figure 1:**
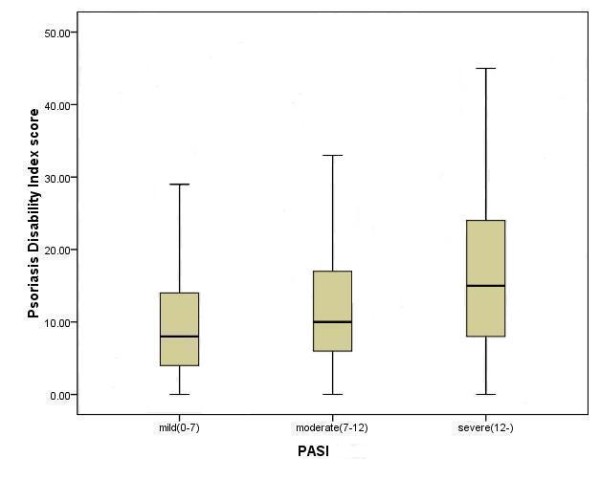
Box plot of the PDI score stratified to severity of psoriasis assessed by the PASI.

According to the PASI score assessed by physicians, there were 348 patients (42%) with PASI <7, 195 patients (23%) with PASI 7–12, and 288 patients (35%) with PASI ≥ 12. Therefore, the subjects were divided into three “severity” groups: mild, moderate, and severe. The PDI score showed significantly different among the three groups (Kruskal-Wallis test*χ*^2^=76.30, *P* < 0.001) and increased with the increasing severity of psoriasis (Figure 1).

### Dimensionality of the PDI

Exploratory factor analysis using an eigenvalue of >1.0 as the criterion resulted in two factors for working and three for nonworking subjects (Table 4), which accounted for 57.3% and 62.3% of the total PDI variance, respectively. All the 15 items were retained when the criterion of highest loading was used, above 0.40 and at least 0.10 stronger than the next. For working subjects, the first factor may reflect working and social disabilities and the second factor may reflect hygienic disability. For nonworking subjects, the first two factors contained the same items as those for the working subjects but item 14 (More smoke/drink), which was extracted separately as the third factor.

**Table 4:** Exploratory factor analysis of the PDI

**Scales**	**Items**	**Patients who were working (n = 607)**		**Patients who were not working (n = 224)**		
		**Factor I** **(eigenvalue = 7.46)**	**Factor II** **(eigenvalue = 1.13)**	**Factor I** **(eigenvalue = 7.08)**	**Factor II** **(eigenvalue = 1.21)**	**Factor III** **(eigenvalue = 1.06)**
Daily activities	1. House/garden work	**.46**	.33	**.51**	.34	.03
	2. Different clothes	-.03	**.80**	.17	**.63**	-.06
	3. Change/wash clothes	.02	**.81**	.04	**.85**	-.05
	4. Hairdresser problem	-.05	**.79**	.32	**.49**	.01
	5. More baths	-.11	**.82**	-.30	**.96**	.07
Work	6a. Time off work	**.83**	-.02	**-**	**-**	**-**
	7a. Inhibit work	**.81**	-.01	**-**	**-**	**-**
	6b. Less activity	**-**	**-**	**.83**	.03	-.05
	7b. Different activity	**-**	**-**	**.70**	.18	-.10
	8. Career affected	**.86**	-.09	**.80**	-.21	.04
Personal relations	9. Sexual difficulties	**.75**	.00	**.87**	-.11	-.06
	10. Social relations	**.73**	.07	**.81**	-.01	.00
Leisure	11. Social activities	**.61**	.23	**.69**	.15	.01
	12. Sport difficulties	**.55**	.25	**.85**	-.07	-.05
	13. Communal changing	**.77**	-.11	**.51**	.01	.41
	14. More smoke/drink	**.62**	-.13	-.06	-.00	**.96**
Treatment	15. Home messy/untidy	**.60**	.22	**.52**	.14	.12

Bold values indicate the largest factor loadings for each item.

## Discussion

As it is increasingly important to assess the impact of psoriasis on the lives of patients, this study adapted an original English and widely used scale PDI to Chinese psoriasis patients. The Chinese version of the PDI was proved to have good reliability and validity by the data presented here.

### Response distribution of the PDI

A moderate percentage of patients were likely to response “Not at all” affected by psoriasis for the work, personal, and treatment subscales of the PDI. Comparing to the large floor effects conducted in a US study (>49% except daily activities), the floor effects of the Chinese version of PDI were smaller while both ceiling effects were small [32]. The mild floor effects might suggest that some items of the PDI provide limited information on the disabilities caused by psoriasis, especially for those with low level of disabilities and severities.

### Reliability and validity of the PDI

The internal reliability estimated by Cronbach’s alpha exceeded 0.7 in the total scale and all subscales of the PDI, which indicated a good reliability of the instrument. Similar results have been found in other language versions of the PDI [32,35,36]. Regarding the validity, excellent and moderate to good correlations with the DLQI and certain subscales of the SF-36 added to available evidence of convergent validity for the PDI. The English and Norwegian versions of the PDI have also proved their convergent validity [32,35]. The results of known groups comparison indicated the discriminate ability of the PDI was good enough to distinguish the patients with different severities of psoriasis, which was consistent with previous studies [32].

### Dimensionality of the PDI

The items of PDI were grouped into five subscales based on common sense when the PDI was developed [15]. However, the original structure could not be confirmed by factor analysis [37], and no consensus exists regarding the number of factors composing this scale. It has been observed that three factors labeled physical, social, and hygienic disabilities respectively could underlie the disabilities construct in the Norwegian version [35]. Our results confirmed the two factors conception of the PDI for the working patients, one concerning work-related disabilities, the other concerning hygiene and embarrassment, which was consistent with the results obtained in a US sample [32]. For nonworking patients, our results indicated a similar factor structure as those for working patients, which was not consistent with the extraction of one-factor structure in the US sample, however [32]. The differences between present results and the results in the US sample might be partly due to differences in sample characteristics. The multidimensionality of the PDI documented by the existing studies could compromise the validity of the total score of PDI when it is used for measurement of impact of psoriasis.

### Strengths and limitations

This study is important as being the first study to evaluate reliability and validity of the Chinese version of PDI based on a relatively large sample of Chinese patients with psoriasis. The results presented here provide reasonable evidence to show that the Chinese version of PDI has good reliability and validity. There are some limitations, however. Because the PDI was administrated once to the subjects, test-retest reliability, responsiveness to change, and minimal clinical important difference (MCID) were not evaluated. The differential item functioning (DIF) among patients with different demographic variables and cultural background was not evaluated. Enrolled in large hospitals only, the study sample can not be regarded as representative of all Chinese patients with psoriasis. Moreover, there were nearly 30% sample subjects suffering other chronic disease. The reliability and validity of the PDI in these patients need further studies.

## Conclusion

The Chinese version of PDI is a reliable and valid instrument and can be used to assess patient-reported impact of psoriasis. It is expected to help improve the QOL assessment of Chinese patients with psoriasis because until now there have been few instruments to measure psoriasis-specific QOL in China. The results are comparable to those of the original English version and translated versions in other countries. Therefore, international cooperative research could use the scale to measure QOL in patients with psoriasis.

## Competing interests

The authors declare that they have no competing interest.

## Author’s contributions

ZH contributed to the conception and interpretation of the statistical analysis, and drafted the manuscript. CL contributed to the design of the study, training interviewers and drafting the manuscript. AO conducted the statistical analysis, contributed the interpretation of data. JF contributed to the conception and design of the study, and revised the manuscript. DW, JD, ZZ, and JY contributed to the acquisition of data. All authors read and approved the final manuscript.
